# Facilitating high throughput collections-based genomics: a comparison of DNA extraction and library building methods

**DOI:** 10.1038/s41598-025-88443-0

**Published:** 2025-02-19

**Authors:** William A Marsh, Andie Hall, Ian Barnes, Ben Price

**Affiliations:** Natural History Museum, Cromwell Road, South Kensington, SW7 5BD London, UK

**Keywords:** Biodiversity, Historical DNA, Museomics, Museum collections, Molecular biology, Zoology, Ecology, Conservation biology

## Abstract

While DNA barcoding methods are an increasingly important tool in biological conservation, the resource requirements of constructing reference libraries frequently reduce their efficacy. One efficient way of sourcing taxonomically validated DNA for reference libraries is to use museum collections. However, DNA degradation intrinsic to historical museum specimens can, if not addressed in the wet lab, lead to low quality data generation and severely limit scientific output. Several DNA extraction and library build methods that are designed to work with degraded DNA have been developed, although the ability to implement these methods at scale and at low cost has yet to be formally addressed. Here, the performance of widely used DNA extraction and library build methods are compared using museum specimens. We find that while our selected DNA extraction methods do not significantly differ in DNA yield, the Santa Cruz Reaction (SCR) library build method is not only the most effective at retrieving degraded DNA from museum specimens but also easily implemented at high throughput for low cost. Results highlight the importance of lab protocol on data yield. An optimised “sample to sequencing” high-throughput protocol which incorporates SCR is included to allow for easy uptake by the wider scientific community.

**Supplementary Information:**

The online version contains supplementary material available at 10.1038/s41598-025-88443-0.

##  Introduction

DNA-based identification is an increasingly principal component of biology, as it allows rapid and accurate species identification from a range of sample types. However, the potential for this approach is limited by the availability of a high-quality reference databases^1,2^. Barcode reference sequence databases need to not only have good sequence accuracy, but also incorporate a wide range of taxa from across the range of candidate species, and contain sequences derived from expertly identified specimens^3^.

Specimens from museum collections are well placed to make an important contribution in this context given that museums are estimated to house more than a billion specimens, covering the vast majority of described species^4–6^.

The potential for museum collections to act as “storehouses of DNA” has been well appreciated for decades^7^, but it is only with the advent of cheaper DNA extraction and sequencing methods, coupled with increased automation, that they are starting to meet that potential^8^. DNA recovered from museum specimens is of lower quality than that found in fresh tissues, and so specialist methods are required to work at scale across multiple taxonomic groups of a range of ages. Of particular importance is that the DNA in collections specimens is highly fragmented^9^, rendering the use of PCR based methods extremely difficult, especially if the required dataset is across a wide taxonomic range^10^.

In order to develop a protocol for DNA barcoding that was compatible with the scale required to fill gaps in the European barcode reference library, suitable for use with museum specimens at low cost, and had the potential for automation to allow high throughput and speed, the performance of two DNA extraction methods ^11,12^, and three library build methods, two of which are commercially available kits, alongside a recently published DIY method (Santa Cruz Reaction;^13^) were tested. Performance was assessed using metrics associated with the quality and quantity of DNA recovered, and each protocol‘s ability to be carried out at scale and at low cost is considered.

## Methods

### Preliminary investigation of DNA extraction methods

Methods previously used on museum specimens can be broadly split into those using precipitation, or those using magnetic beads or silica columns to capture the DNA. With the overarching goal to develop a low-cost high-throughput method we did not test a commonly used ancient DNA (aDNA) specific DNA extraction method^14^, given (i) large volume centrifugation (reducing potential for scaling and high throughput) and (ii) relatively expensive large volume silica columns. However binding buffer D^14^ was used with silica beads following^15^, with beads offering similar DNA retention but allows for upscaling. We did not test ethanol/isopropanol precipitation methods due to the large volume centrifugation steps which require expensive equipment and cannot be scaled up sufficiently for high throughput.

Initial tests used Ultra Low Range Ladder (ThermoFisher cat. # 10597012) which has 10–300 bp bands, reflecting the size distribution typical for museum specimens^9,16^. For each tested method mock lysate was produced using 0.5uL of ladder (250ng input DNA) and 90uL lysis “Buffer C”^17^; 200mM Tris pH8,25mM EDTA pH8,0.05% (vol/vol) Tween 20,0.4 mg/ml Prot. K), excluding the Proteinase K. Each method was then followed as published with four replicates, and the resulting DNA extract was quantified using a Qubit 4 Fluorometer (dsDNA assay kit), and a Tapestation 4200 (Agilent) using D1000 tapes. The best performing column and magnetic bead methods, as measured by percentage recovery of input DNA ≥ 35 bp, were further refined with ULR ladder and then taken forward for testing with museum specimens.

## Specimens

Two species with high quality reference genomes were selected covering a range of ages: four specimens of the beetle *Rhagonycha fulva* collected between 1953 and 2011: NHMUK011517159 (coll. 1953), NHMUK011517162 (coll. 1972), NHMUK011517258 (coll. 2005), NHMUK014422990 (coll. 2011); and one specimen of the bumblebee *Bombus pascuorum* collected in 1964 (NHMUK014564519) were sourced from the collections of the Natural History Museum, London (NHMUK). We focus on Insecta species, given (i) their utility as ecosystem health indications, (ii) their global prevalence and high species diversity, (iii) their ubiquity in natural history collections, and (iv) the prediction that genomic studies incorporating insect species will increase over the next decade^5^. Number of samples incorporated was not statistically predetermined and was instead dependent on the availability of samples and feasibility of performing the experiment. Prior study has suggested that DNA preservation is largely consistent across insect specimens under the same curatorial stewardship^9^, meaning numerous replicates were not required in this instance. This limited sample size also ameliorated ethical issues associated with the sampling of museum specimens solely for developmental purposes, in line with institutional guidelines.

For each insect specimen, one leg (bumblebee) or two legs (beetles) were detached with sterile forceps and lysed overnight at 56 °C in 90ul of lysis buffer C^17^. The lysate was then divided into two aliquots of 45ul, with the first aliquot processed following DNA extraction method 1 and the second aliquot processed following DNA extraction method 2 below:

## DNA extraction methods

### DNA extraction method 1 – Rohland (R)

Lysate was extracted as per^12^ DNA purification option B, with a ratio of 10:1 binding buffer D and the following modifications: 45ul of lysate was processed following the method for 150ul lysate. Beads were dried for approx. 5 min, until visibly dry.

### DNA extraction method 2 – Patzold (P)

Lysate was extracted using a Monarch PCR & DNA Clean-up Kit (New England Biolabs) via a modified version of ^11^. Lysate was made up to 50ul with ultra-pure water. 100ul of DNA clean up binding buffer was added, followed by 300ul 100% ethanol. After gentle mixing by flicking, the total volume was passed through a spin column, 700ul at a time. Columns were washed twice with 500ul of wash buffer. Supernatant was discarded after each spin.

Columns were centrifuged for 1 min to dry the membrane then transferred to clean 1.5 ml low bind Eppendorf tubes. Elutions were 2 × 15ul of elution buffer with 1 min RT incubation.

All centrifugation steps were 30s at 16,000 g.

DNA was then quantified using Qubit dsDNA HS reagents and assessed using an Agilent Tapestation with High Sensitivity D1000 ScreenTapes.

## Library build methods

Illumina libraries were prepared from each DNA extraction via the following methods:

### Library method 1: NEB next ultra II library preparation kit (New England Biolabs) - NEB

The manufacturer’s protocol *NEBNext® Ultra™ II DNA Library Prep Kit for Illumina® for use with NEBNext Multiplex Oligos for Illumina (Unique Dual Index UMI Adaptors DNA Set 1, NEB #E7395*) Steps 1 to 3b were followed with the following exceptions:

- Half volumes of all reagents were used.
- All SPRI bead cleans were 1.2x to best retain small fragments.

Post-ligation clean-up, libraries were amplified using P5 and P7 Illumina primers and AmpliTaq Gold™ mastermix as per the manufacturer’s instructions. AmpliTaq Gold™ was used instead of NEB Next Q5 Mastermix as it is uracil tolerant, following consultation with NEB tech support. Cycle number was determined by input concentration as recommended in the NEB Next Ultra II protocol. Cleaned libraries were assessed via Tapestation 4200 (Agilent) with D1000 tapes. Three samples contained large amounts of adaptor-dimer so were cleaned with 1x SPRI beads.

### Library method 2: xGen™ ssDNA & low-input DNA library preparation kit - IDT

Libraries were prepared following the manufacturer’s instructions with one exception: indexing PCR was performed with AmpliTaq Gold™ mastermix as per the manufacturer’s instructions. AmpliTaq was used instead of the reagents supplied with the kit as it is uracil tolerant, following consultation with IDT tech support. Cycle number was determined by input concentration as recommended in the xGen protocol. Cleaned libraries were assessed via Tapestation 4200 (Agilent) with D1000 tapes.

### Library method 3: Santa Cruz Reaction (SCR) - SCR

Libraries were prepared as per^13^ with the following modifications;

i) Indexing PCR cycle number was estimated from ng DNA input, not using qPCR, as:

**Table:** 

DNA (ng)	PCR cycles
2–4.9	10
5–19.9	8
20- 29.9	6
30–41	4

ii) The entire purified product from SCR library build was indexed, not just 2ul.

Indexing PCR products were cleaned using 1.2x QuantBio SparQ beads as per manufacturer’s protocol “post PCR amplification clean-up 1 C”. Cleaned library DNA concentration was assessed via Tapestation 4200 (Agilent) with D1000 tapes. Three samples showed no discernible DNA content and indexing PCR was repeated for an additional 4 cycles. The commercial kit available most similar to SCR (SRSLY™ from Claret Biosciences) was not included here, due to its similar performance to SCR^13^ and increased cost (~ 10x more expensive).

All 36 libraries were normalised and pooled (equimolarity calculated using outputs from both Qubit and Tapestation) before being sequenced on a NextSeq500 Mid output PE 2 × 75 kit at the Natural History Museum sequencing facility.

### Computational analysis

Samples were demultiplexed using blc2fastq2. Adapters were trimmed and paired end reads collapsed from demultiplexed raw fastq files using Adapterremovalv2.0 (^18^; —mm 3 —minlength 30). For IDT treatment samples,10 bases were trimmed off the 5’ and 3’ ends of each read following the manufacturer’s protocol. Only collapsed reads were retained for alignment.

Alignment was performed using bwa v 0.7.17^19^ with a random seed and an initial low quality filter (-l 1024 -q 10) to the relevant species mitochondrial and nuclear reference genomes. Genomes used were accessed from NCBI (assession numbers GCA_905340355.1, HG996561, GCA_905332965 and HG995285) and were all recently published assemblies stemming from the Darwin Tree of Life Project (*R. fulva:*^20^; *B. pascuorum:*^21^). Output sam files were converted to bam files using samtools^22^, before reads were sorted, quality filtered (QC ≥ 30) and duplicated reads removed (see Supp. T1 for alignment details).

To allow for comparisons across specimens, library and DNA extraction methods, several summary statistics were calculated. Endogenous content (Q30 unique mapped reads/total sequenced reads), duplication rates (Q30 unique mapped reads/Q30 mapped total reads) and collapsed read proportions (total collapsed reads/total sequenced reads) were calculated from the read count outputs of samtools. Average read length and GC content for each sample were obtained using Qualimap^23^, and samtools stats $i | grep ^RL | cut -f 2 used to obtain read length distributions. Preseq^24^ lc_extrap was used to calculated library complexity using unfiltered sorted bam files.

Statistical analysis and data visualisation was performed in R v4.1.1 (R Core Team, 2021).

## Results

Normalised costs for each DNA extraction and library build method can be found in Supplementary Table 2. In short, there is little cost difference between the two extraction methods compared. SCR is 15x cheaper than the next cheapest method (IDT), although we did not consider the potential to use smaller reagent volumes than recommended for each kit.

### Wet lab

#### Testing DNA extractions

Based on Qubit quantification of the ULR ladder the best performing silica column method was^11^, 2020 which recovered an average of 74% input DNA (range: 61 − 89%), followed by^17^  which recovered an average of 67% input DNA (range: 60 − 77%).

The best performing magnetic bead method was^12^ which recovered an average of 45% input DNA (range: 41 − 48%), followed by^25^ which recovered an average of 38% input DNA (range: 37 − 40%).

While silica columns recover more total DNA tapestation reports suggest the much higher recovery from silica columns is due to the recovery of 10 bp and 20 bp fragments which together account for 40% of the input DNA in the ULR ladder.

### Sequence data

#### DNA extraction comparison

**Fig. 1:**
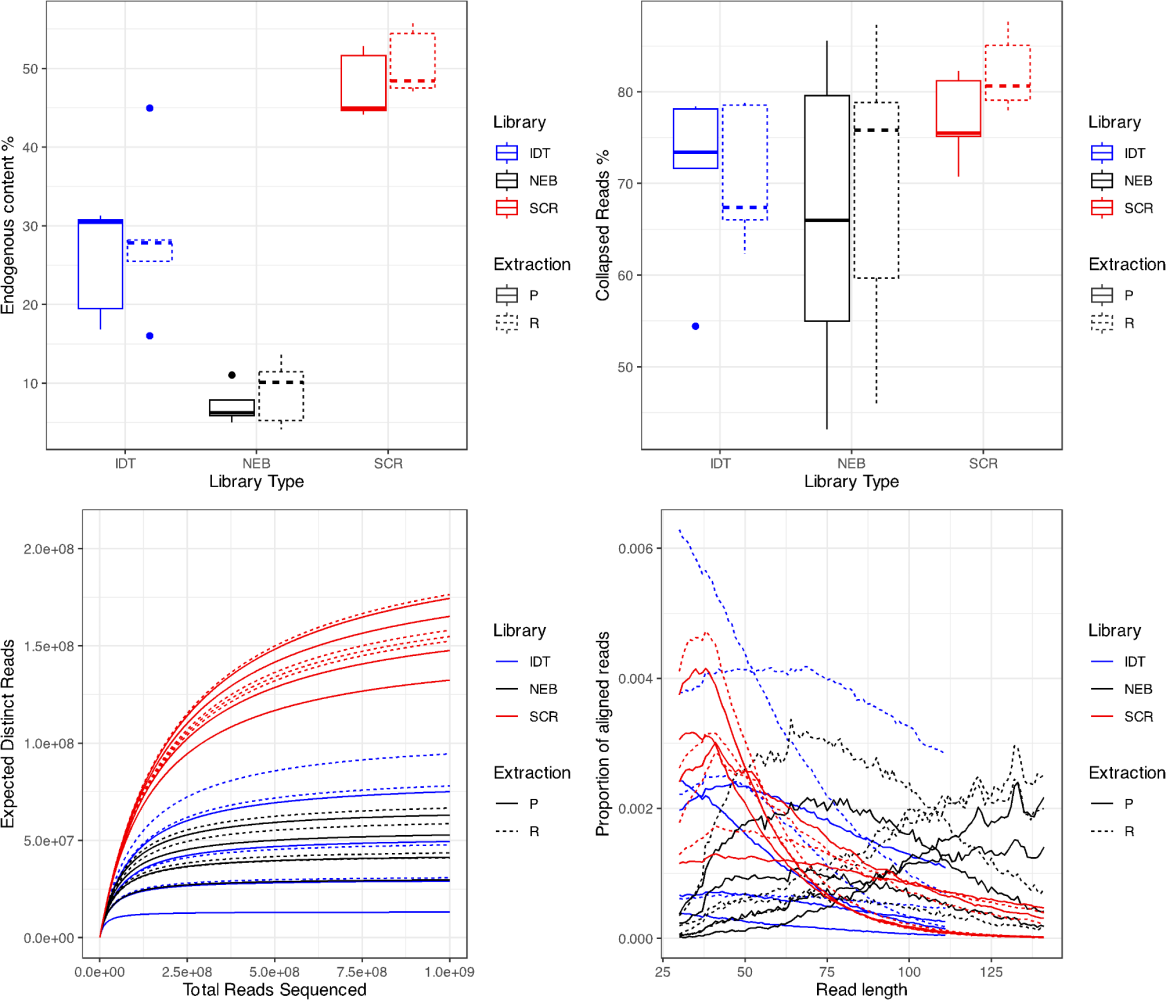
The effect of DNA extraction and library building protocols on (**a**) endogenous content; (**b**) collapsed read proportion; (**c**) library complexity (as calculated using preseq); and (**d**) aligned q30 unique read length distribution. See Supplementary Table 1 for detailed alignment statistics.

Of the two DNA extraction methods carried forward to sequencing, no significant difference is identified in terms of endogenous content, aligned q30 unique read length distribution, library complexity (calculated using preseq), and collapsed read proportion (Fig. 1; Table 1, Supplementry Table 1)

### Library build comparison

Library building protocol was found to significantly impact all summary statistics calculated, with the Santa Cruz Reaction^13^ showing the best performance of the three treatments (Fig. 1; Table 1, Supplementry Table 1).

Endogenous content was found to be significantly higher in libraries built using the SCR method (Fig. 1(a); ANOVA: *p* < 0.01, R^2^ = 0.745).

Read length was found to differ significantly across library building methods (*p* < 0.01; R^2^ = 0.773; Table 1). Read length distributions for each sample can be seen in Fig. 1(b), with samples subjected to the SCR showing read length distribution indicative of a higher propensity for short, degraded DNA to be sequenced when compared with the profiles of both IDT and NEB. Results for IDT and NEB indicate more longer reads are incorporated into built libraries and subsequently sequenced. Specimen was found to significantly influence read length, indicating that the preservation history and DNA fragmentation profile that is unique to each specimen impacts the read length of sequenced DNA regardless of DNA extraction or library treatment.

Library complexity was found to differ across library building methods (Fig. 1c), with SCR libraries showing greater complexity (number of predicted unique reads) than both IDT and NEB methods. DNA extraction method has no effect on library complexity.

The proportion of collapsed reads was found to differ significantly across library building methods (Fig. 1d; ANOVA: *p* < 0.05; R^2^ = 0.221), with samples subjected to SCR showing a greater number of collapsed reads than both IDT and NEB methods. DNA extraction method has no impact on the proportion of collapsed reads.

**Table 1:** P values for nested anovas in the form: Dependant variable = Sample(factor = Library build) + Sample(factor = Extract). Significant results highlighted in bold .

Independent variables	Endogenous mapped reads	Endogenous mapped bases	Duplication rate	Read length	Proportion of collapsed reads
Sample	0.524	0.525	0.304	9.86E-05	0.113
Library build method	3.21E-08	8.49e-08	5.74E-09	3.26E-09	0.0326
DNA extraction method	0.734	0.720	0.862	0.999	0.9501

###  At scale

Following on from the results of this study, the protocol developed and shared here has successfully been implemented at scale by authors. Over 40 96 sample well plates have been run over the course of a year, resulting in the generation of over 300 billion paired end reads (20x Illumina X Plus 25B 2 × 150PE flow cells, and 5x Illumina X Plus 10B 2 × 150PE flow cells). Steps have also been taken to consider and ameliorate potential issues associated with ancient DNA^26^and cross contamination. These steps included (i) the separation of pre- and post- PCR steps to minimise PCR contamination during extraction, (ii) the testing for cross contamination within a well plate using alternating positive and negative controls, and (iii) a reduction in binding buffer volume that reduces the potential for any cross contamination during extraction, but has no impact on efficiency (detailed in the SI). Although the use of an aDNA specific clean lab for all pre-PCR steps would be optimal, the high throughput required and the limited PCR contamination seen both within and between samples reduces its necessity in this instance. However, when extremely degraded, precious or ancient samples are incorporated, it remains essential to perform all pre-PCR steps in a dedicated ancient DNA laboratory^27^.

## Discussion

The Santa Cruz library build Reaction (SCR) is found to generate indexed DNA libraries that allow significantly more of the DNA endogenous to the museum specimen to be sequenced and aligned (Fig. 1). The complexity of SCR libraries is greater than other tested library building methods, with the read length distribution indicating that the SCR method incorporates more short reads (indicative of degraded DNA) into indexed libraries. IDT shows intermediate performance, whilst NEB performs the worst of the three library building methods. DNA extraction method is not seen to influence the quality and quantity of sequence data produced, and no relationship between DNA extraction and library build methods is identified.

At high throughput, the column-based DNA extraction method^11^ is both more costly and more time consuming^28^ than the bead based^12^ method. As such^12^, is preferential for low-cost high throughput activities.

The performance of SCR here is not surprising, given it was developed to incorporate short deaminated reads that are commonly found in degraded museum specimens^13^. The method has previously been shown to offer similar performance to the aDNA specific ssDNA2.0^29^ and BEST^30^ library build methods, with the performance of SCR only seen to be drop-off when input DNA mass is extremely low and deamination is prevalent^13^, properties that are not widely seen in historical museum specimens. The worst performing library build method tested in this study is not explicitly designed for degraded DNA from museum specimens but instead is aimed at DNA from modern samples, with the expected DNA fragmentation and deamination identified previously in museum specimens likely limiting performance^9,31^. Results serve to highlight the importance of considering the expected DNA preservation of specimens prior to deciding on wet lab protocols.

The ability to implement the SCR method rapidly, cheaply and at a high throughput is enabled from the limited cost of reagents and short protocol (Supplementary Table 2). Further, it is seen to outperform the other high throughput library build methods tested here. Given the extensive time and effort taken to increase DNA yields whilst reducing cost, we present a complete step-by-step protocol “from sample to sequencing” in the supplementary text, allowing for quick and easy implementation by other researchers looking to incorporate museum specimens into their research. We clearly demonstrate that this optimised protocol can be easily and efficiently upscaled with a single lab technician producing 760 libraries per month using SCR with minimal automation, generating over 300 billion DNA sequence reads over the last year.

## Conclusion

The Santa Cruz Reaction (SCR) library build^13^ is shown to outperform off-the-shelf library build methods. These results, alongside the speed and cost of the method, indicate that SCR should be strongly considered for use in future large scale museomics projects where sequence data of high quantity and quality at low cost is required . An optimised “sample to sequencing” high throughput protocol which incorporates a bead-based DNA extraction and SCR is provided.

## Electronic supplementary material

Below is the link to the electronic supplementary material.

Supplementary Material 1

Supplementary Material 2

## Data Availability

Sequence data supporting the findings of this study are deposited on Zenodo: https://zenodo.org/records/13642777.
