# Publisher Correction: Facilitating high throughput collections-based genomics: a comparison of DNA extraction and library building methods

**DOI:** 10.1038/s41598-025-94879-1

**Published:** 2025-03-19

**Authors:** William A. Marsh, Andie Hall, Ian Barnes, Ben Price

**Affiliations:** Natural History Museum, Cromwell Road, South Kensington, London, SW7 5BD UK

Correction to: *Scientific Reports* 10.1038/s41598-025-88443-0, published online 19 February 2025

The original version of this Article contained an error in the legend of Fig. 1, where the descriptions of panels (**b**) and (**d**) were switched.

“The effect of DNA extraction and library building protocols on (**a**) endogenous content; (**b**) aligned q30 unique read length distribution; (**c**) library complexity (as calculated using preseq); and (**d**) collapsed read proportion. See Supplementary Table 1 for detailed alignment statistics.”

now reads:

“The effect of DNA extraction and library building protocols on (**a**) endogenous content; (**b**) collapsed read proportion; (**c**) library complexity (as calculated using preseq); and (**d**) aligned q30 unique read length distribution. See Supplementary Table 1 for detailed alignment statistics.”

Furthermore, Supplementary Material file 1 published with the original version of this Article was a previous version. Due to an error during production the incorrect file was assembled for publication.

The original Article and the accompanying Supplementary Material file 1 have now been corrected.

