# Supplementary material for: Facilitating high throughput collections-based genomics: a comparison of DNA extraction and library building methods
Source: Sci Rep. 2025 Feb 19;15:6013. doi: 10.1038/s41598-025-88443-0 (PMC11839992; doi:10.1038/s41598-025-88443-0)
Supplement: Supplementary file 1 — Supplementary Material 1 [file 41598_2025_88443_MOESM1_ESM.pdf]

Supplementary Information

Protocol refinements:

Refining the magnetic bead method - throughput

To increase potential throughput manual bead extraction was compared to robotic extraction on a KingFisher FLEX (ThermoFisher) using eight independent lysates of pinned insect legs (Odonata), split equally between the manual and robotic method. Total DNA recovered was measured using the Qubit 4 Fluorometer with BR dsDNA assay kit, and a Tapestation 4200 (Agilent) using D1000 tapes.

Refining the magnetic bead method - cost

Subsequent tests used the KingFisher APEX (ThermoFisher) which uses the same approach as the FLEX and includes in-built UV sterilization between runs. Several silica magnetic beads were tested given the high cost of G-Biosciences magnetic beads (VWR cat. # GENO786-915: £526.80 for 5mL = £1.05 per sample) used by (Rohland and Hofreiter, 2007).

Tested beads included: MagAttract Suspension-G (Qiagen cat. # 1026901: £234.00 for 13mL = £0.18 per sample); SeraSil 400 (VWR cat. 29-3573-71: # £572.40 for 60mL = £0.10 per sample); SeraSil 700 (VWR cat. 29-3573-74: £477.60 for 60mL = £0.08 per sample). To reduce costs and ensure the total volume of lysate and binding buffer could fit within a 96 deep-well plate, the volume of binding buffer was optimised by comparing recovery of ULR ladder with 5:1, 8:1 and 10:1 binding buffer:lysate ratios tested. A reduction of lysate volume to 45ul and a subsequent 1:10 ratio of binding buffer (450ul) showed no reduction in extraction performance, whilst allowing for easy upscaling with minimal evidence for contamination.

To further reduce costs a DIY wash buffer (10mM Tris pH8, 80% (vol/vol) ethanol) was compared to buffer PE (Qiagen cat. # 19065), based on an earlier comparison with fresh samples (pers. comm.: Kevin Beentjes, Naturalis Biodiversity Center, Leiden). The resulting protocol is available here: https://doi.org/10.17504/protocols.io.4r3l27ebxg1y/v2.

Refining SCR library builds

The best performing library build, SCR, was further optimised after the lab work for this study had been completed. Rather than estimating the number of PCR cycles from the mass of input DNA, a qPCR step was added to ensure the optimal number of PCR cycles for each sample was chosen. Samples can be batched for CT values of –1 to +3, following Kapp et al 2021. To save time and reduce costs, reaction clean-ups (post adapter ligation and post indexing PCR) were replaced with a SPRI bead clean up following (Rohland and Reich, 2012). Tier 5 of SCR was replaced with the Spotlight method (Nguyen et al., 2023).

These modifications are included in the complete “sample to sequencing” protocol detailed below.

References:

Kapp, J.D., Green, R.E., Shapiro, B., 2021. A Fast and Efficient Single-stranded Genomic Library Preparation Method Optimized for Ancient DNA. Journal of Heredity 112, 241–249. https://doi.org/10.1093/jhered/esab012

Nguyen, R., Kapp, J. D., Sacco, S., Myers, S. P., & Green, R. E. (2023). A computational approach for positive genetic identification and relatedness detection from low-coverage shotgun sequencing data. Journal of Heredity, 114(5), 504–512. https://doi.org/10.1093/jhered/esad041

Rohland, N., Hofreiter, M., 2007. Ancient dna extraction from bones and teeth. Nat Protoc 2, 1756–1762. https://doi.org/10.1038/nprot.2007.247

Rohland, N., Reich, D., 2012. Cost-effective, high-throughput DNA sequencing libraries for multiplexed target capture. Genome Res 22, 939–946. https://doi.org/10.1101/GR.128124.111

Full "Sample to sequencing" protocol associated with Marsh et al., 2024:

Extraction

Protocol here: https://dx.doi.org/10.17504/protocols.io.4r3l27ebxg1y/v2

Buffer preparation

1. **Lysis buffer C** *200mM Tris pH8, 25mM EDTA pH8, 0.05% Tween 20, 0.4mg/ml PK*
   To make 10 mL mix together: 7295 µL molecular grade water, 2000 µL 1M Tris pH8, 500 µL 0.5M EDTA (pH 8), 200 µL Proteinase K (20mg/ml), and 5 µL Tween 20.This is sufficient for 1 extraction plate of 95 samples and 1 negative control (90 µL each).
2. **Binding buffer D** *5 M guanidine hydrochloride, 40% (vol/vol) 2-propanol, 0.12 M sodium acetate and 0.05% (vol/vol) Tween 20.* In a glass or plastic bottle, weigh 124.2 g of guanidine hydrochloride and fill up with molecular water to 150 mL . Heat briefly in a microwave until the buffer is warm to the touch and shake until the salt is fully dissolved. Add 104 mL of 2-propanol, 10.4 mL of 3M sodium acetate buffer solution (pH 5.2) and 130 µL of Tween 20. This is sufficient for 6 extraction plates of 95 samples and 1 negative control (450 µL each). Store in a fridge for up to 4 weeks. Seal the bottle with Parafilm to avoid evaporation.
3. **Wash buffer** *10mM Tris HCl 80% EtOH (vol/vol).* To 5 mL 1M Tris-HCl, add 412 mL 97% Ethanol and 83 mL molecular grade water. This makes 500 mL of wash buffer, sufficient for 6 extraction plates of 96 wells (750 µL each). This buffer can be stored at room temperature for at least 1 year.
4. **Elution Buffer** To make 50 mL of elution buffer, combine 49.4 mL of molecular water, 500 µL of 1 M Tris-HCl (pH 8.0), 100 µL of 0.5 M EDTA (pH 8.0) and 25 µL of Tween 20. This is sufficient for 10 96-well extraction plates 50 µL each). This buffer can be stored at room temperature for at least 1 year.

Silica bead preparation

MagAttract Suspension G magnetic beads (Qiagen: 1026901) The original Rohland protocol washes the beads before use, but we have found unwashed beads work just as effectively.

Manual protocol

Tissue can be added to the plate/tubes either before or after the addition of lysis buffer, however tissue from dried specimens (e.g. pinned insects) should be added to wells/tubes with lysis buffer to prevent static displacement. If tissue was previously stored in ethanol this should be dried off before lysis.

1. Add 90 µL of lysis buffer C to each sample tube / well. Volume can be adjusted to ensure coverage of tissue, however be mindful that 5x volume of binding buffer is required further down the protocol.
2. Incubate overnight at 56 °C on a shaking incubator. The longer the incubation the more the tissue / specimen is digested. Shorter incubations are recommended for better voucher recovery with whole body lysis.
3. Pipette lysate into a new tube / deep-well plate. If tissue / voucher is to be recovered then add appropriate volume of 80% ethanol to cover sample in original tube / well.
4. Add 450 µL of binding buffer D (= 5x volume of lysate) to lysate in tube / deep-well plate.
5. Add 10 µL of silica magnetic bead suspension to lysate and binding buffer.
6. Vortex for 5sec.

7. Rotate (mix) the tubes / plates for 15min at room temperature.
8. Spin briefly in a microcentrifuge to collect the suspension at the bottom and place them on an appropriate magnet.
9. Once solution clears pipette off the supernatant and discard.
10. Remove from the magnet, add 250 µL of wash buffer and vortex for 8sec.
11. Spin briefly to collect the suspension at the bottom and place back on the magnet.
12. Repeat wash (steps 9 - 11) twice for a total of three washes.
13. Aspirate any remaining drops of liquid and dry the beads at room temperature by leaving them on the magnet with open lids. Usually a few minutes is sufficient.
14. Remove from the magnet, add 50 µL - 100 µL of elution buffer depending on original sample size and anticipated DNA recovery.
15. Vortex until all beads have been resuspended and then briefly spin down.
16. Incubate for 2min at room temperature.
17. Place back on the magnet, wait until the solution clears and transfer the supernatant to a fresh tube / plate.

Proceed to library preparation or freeze extracted DNA.

Automated protocol

The manual protocol has been tested on the Kingfisher Flex and Apex platforms. The protocol starts after step 5 above, i.e. prepare the deep-well plate with 90 µL lysate + 450 µL binding buffer D and 10 µL silica beads.
The kingfisher protocol uses onboard "tip mixing" rather than vortexing / mixing to resuspend beads and skips all centrifugation steps.

SCR and Spotlight library preparation protocols combined

Prepared by Alyssa Paul, Carla Gustave, Owain Powell and Ben Price

This protocol is a combination of Santa Cruz Reaction (SCR) and "Spotlight" used at ½ original published volume for library building museum specimens. Please cite the original publications if using this method. SCR: https://doi.org/10.1093/jhered/esab012; Spotlight: https://doi.org/10.1093/jhered/esad041

Protocol choice by quantity of dsDNA:

Protocol	dsDNA* (ng) (half volume)	P5 (µM)	P7 (µM)	SSB (ng/µL)
SCR tier 1	15 - 37	12	6	328
SCR tier 2	8 - 15	6	3	164
SCR tier 3	3.5 - 8	3	1.5	82
Spotlight	0 – 3.5	2	0.4	76

Adapter Preparation

NOTE: (SCR adapter/splint preparation method is used even in spotlight protocol, but CUTSMART buffer is used to dilute instead of adapter dilution buffer all round)

1. Resuspend all oligonucleotides to 100 µM using TE buffer.
2. Add the following components to 0.2 mL tubes:

**P7**	
Water	30.6 µL
100 µM P7_**adapter**	6 µL
100 µM P7_**splint**	8.4 µL
10X T4 RNA Ligase Buffer	5 µL

**P5**	
Water	30.6 µL
100 µM P5_**adapter**	6 µL
100 µM P5_**splint**	8.4 µL
10X T4 RNA Ligase Buffer	5 µL

3. In a thermocycler with heated lid (105 °C), hybridize the adapters and splints by incubating at 95 °C for 1 minute before ramping down to 10 °C at 0.1 °C per second.
4. Store hybridized P5 (12 µM) and P7 (12 µM) adapter stock solutions at -20 °C. Freeze / Thaw no more than 3x.

Diluting Adapters:

- Dilute 10X CutSmart buffer to 1X (1:10d).
- Dilute the annealed P5 adapter/splint from 10 uM to **2 mM or 6, 3, 1.5 mM** using 1X CutSmart buffer.
- Dilute the annealed P7 adapter/splint from 10 uM to **0.4 mM or 6, 3, 1.5 mM** using 1X CutSmart buffer.
- Store annealed P5 and P7 adapters separately at -20C. **Freeze/thaw dilutions no more than 3x**.

Workflow:

1. **Combine the extracted DNA and SSB**
    1.1 Combine the following in a 0.2 mL PCR strip tube (SCR based on tier)
    1.2 Vortex 30s, and spin down x2.

Spotlight	
DNA extract	10 µL
**76 ng/mL** ET SSB	1 µL

SCR	
DNA extract	10 µL
**[328, 164, 82 ng/mL]** ET SSB	1 µL

2. **Denature the input DNA**

    2.1 Heat DNA/SSB's for 3 min at 95°C in a thermocycler with a 105°C heated lid.
    2.2 Immediately cold shock in a cooling block or on ice for 2 min.
    2.3 Spin down reactions for 5 seconds then place back on ice.

3. **Add Adapters**

    3.1 While the reactions are on ice or in a cooling block, add the following:

Spotlight	
**2 uM** annealed P5 adapter/splint	0.5 µL
**0.4 uM** annealed P7 adapter/splint	0.5 µL

SCR	
**[12, 6, 3 uM]** annealed P5 adapter/splint	0.5 µL
**[6, 3, 1.5 uM]** annealed P7 adapter/splint	0.5 µL

- add to strip tubes then use multichannel to add to plates
- can combine P5 + P7 but must be quick to add DNA
- do not vortex the combined adapters very vigorously or will damage
- <u>do not store</u> combined adapters

4. **Add Reaction Mix**
    4.1 While the reactions are on ice or in a cooling block, add **13 µL** of reaction mix to the plates.

    4.2 Foil seal plates and pulse vortex for 10 seconds, spin down (~7s) x2
    crucial to mix well after adding reaction mix

**Reagent**	**Concentration**
PEG 8000	20 %
Tris-HCl	50 mM
MgCl	10 mM
DTT	10 mM
ATP	1 mM
T4 DNA ligase	25 U/uL
T4 PNK	0.125 U/uL

- add reaction mix to strip tubes then add with multichannel
- solution quite viscous so pipette slowly and pipette mix to get everything out of the tips

5. Incubate Reactions

5.1 Incubate reactions for 1 hour in a pre-heated thermocycler at **37°C**, with lid set to **50°C**.

6. Clean Spotlight Reactions

6.1. Spin down reactions for 5s

6.2. Add 27.5 µL EBT buffer

6.3. Add 37.5 µL 18% PEG SPRI

6.4. Mix and incubate for 10 minutes at RT.
6.5. Continue performing the SPRI clean as normal (See section Bead Clean with Stamp Magnet Protocol).
6.6. Elute in 22 µL of EBT buffer.

** Safe Stop Point - can stop here and store reactions in fridge until ready for qPCR**

7. qPCR

Quantitative PCR is recommended to inform the optimal cycle number for each sample during index PCR.

7.1 Prepare the following reaction for each library:

	1 reaction (per well)	1x 96 well plate (105 rxns incl. 2x2 neg controls)	88 +2 = 90 rxn ~95rxns
LUNA SYBR qPCR Master Mix (K0221)	10 µL	1050 µL	950 µL
H2O	8 µL	840 µL	760 µL
10 µM Primer IS7	0.5 µL	52.5 µL	47.5 µL
10 µM Primer IS8	0.5 µL	52.5 µL	47.5 µL
Library	1 µL	1 µL	1 µL
Total	20 µL		

*NOTE: IS7/8 primers are at 100 µM, can dilute with water to make up 10 µM

7.1.1 Leave last column (12) blank and prepare last column samples in a separate strip tube. Only use two wells as the negative.

<u>7.2 qPCR cycle conditions:</u>

- 95°C for 10 minutes
- 40 cycles of the following:
  - 95°C for 30 seconds
  - 60°C for 30 seconds
  - 72°C for 30 seconds
- Measure the fluorescence at the end of each extension step.

7.2.1 qPCR instrument conditions:

- 96 wells
- Quantitation – comparative Cr
- SYBR Green
- Standard 2-hour run

** NB tick all as sample 1 after selection (box under U/N) or will not show in results**

- Optimal cycle number for index PCR = CT value +1
- Safe range for batching libraries = CT value -1 to CT value +1 (but can also batch between -1 to +3 if necessary)

8. Index PCR

Optimal cycle number can be determined by qPCR (see step 7). Can batch reactions together based on CT values.

8.1 Prepare the following reaction for each library:

Amplitaq Gold 360 Master Mix	25 µL
20 µM i5 index	2.5 µL
20 µM i7 index	2.5 µL
Library	20 µL
	50 µL

8.2 Cycle the reactions in a thermocycler using the following conditions:

- 95°C for 10 minutes
- Library specific number of the following cycle conditions:
    - 95°C for 30 seconds
    - 60°C for 30 seconds
    - 72°C for 60 seconds
- Final extension of 72°C for 7 minutes

Optimal cycle number can be determined by qPCR (see step 7)

9. Clean Index PCR Reaction

- A 1.2x bead clean ie. If 50 µL product then 60 µL beads.

9.1 Add **60 µL** of SPRI (no EBT) and perform the SPRI clean as normal, elute in **22 µL** of EBT buffer (See section Bead Clean with Stamp Magnet Protocol).

9.2 Samples ready for QC and sequencing

- Use Tapestation for post-indexing QC with region analysis to calculate library vs dimer.

Bead Clean with Stamp Magnet Protocol:

(Step 6/9 of Library Prep protocol)

NOTES:

- Use Kingfisher plates (wide)
- Wash Plate: Fill 2x plates with freshly prepared 80% ethanol (200-250 uL) per well (~50 mL)
- Elution Plate: Fill 1x plate with **22 µL** of EBT (~2.2 mL)

1. Transfer samples (~24-26 uL) into wide well Kingfisher plates
2. Add EBT and 18% PEG SPRI beads to sample.
    a. Vortex and incubate for 10 minutes at RT

Spotlight	
EBT buffer	35 µL
18% PEG SPRI	60 µL

SCR	
EBT buffer	27.5 µL
18% PEG SPRI	37.5 µL

3. Add the tip comb to the magnet, add the magnet to the plate and let the beads concentrate until the solution is clear (~3 mins).
   *NB. Keep the remaining supernatant in case the bead clean does not work
4. Transfer the magnet with the beads into the first ethanol wash plate
    a. Move the magnet up/down/side-to-side for about 30 seconds
    b. Move the magnet into the second wash plate and repeat the above steps.
5. Remove the magnet from the ethanol washes and let the excess ethanol dry off (~4 mins). Ensure the beads do not over-dry.
6. Place the magnet into the EBT solution, quickly release the comb from the magnet & mix the beads with the EBT using the comb.
    a. Incubate for 5 minutes at RT
7. Place the magnet back into the plate and let the beads concentrate (~1-2 minutes). Discard the comb with the beads and KEEP the ELUANT.

Preparing SPRI Solution:

1. Add 9 grams of PEG-8000 powder to a 50mL tube.
2. Add the following to the tube:
    - 10 mL of 5M NaCl,
    - 500 uL 1M Tris-HCl
    - 100 uL 0.5M EDTA
3. Add UltraPure water to the 50mL tube up to ~49mL.
4. Cap the 50mL tube, then shake or rotate until PEG has completely dissolved.
5. Add 27.5 uL of Tween-20 to the 50mL tube.
6. Wash Beads:
    - 1.1 Mix SpeedBeads until all beads are in suspension.
    - 1.2 Transfer 1mL of SpeedBeads to a 2.0 mL Eppendorf tube.
    - 1.3 Place tube on a magnetic rack and allow beads to fully concentrate, ~1 min.
    - 1.4 While on the magnetic rack discard the supernatant.
    - 1.5 Add 1mL of TE (10mM Tris, 1mM EDTA) to the tube.
    - 1.6 Cap tube and remove from the rack, then fully resuspend the beads by vortexing.
    - 1.7 Place tube back on the magnetic rack and allow beads to fully concentrate, about 1 min.
    - 1.8 While on the magnetic rack discard the supernatant.
    - 1.9 Repeat steps 5-8 for a second wash.
    - 1.10 Add 1mL of TE to the tube, resuspend by vortexing, then place in a non-magnetic rack.
7. Add the 1mL of washed SpeedBeads to the 50mL tube.
8. Fill with UltraPure water to the 50mL line of the 50mL tube.
9. Gently shake until SpeedBeads are mixed. Wrap tube in foil and store in the dark at 4°C.
